# In Memory of J. E. Kintz

**Published:** 1887-05

**Authors:** 


### IN MEMORY OF J. E. KINTZ.

It seems that Death truly "loves a shining mark," and why so many in the flush of life's opening day, who give promise of being a blessing to their friends and the world, are taken away by an untimely death will remain a mystery until God gives the solution in the light of Eternity.

Mr. J. E. Kintz was born near Sunset, Perry County, Ohio, September 20, 1861, and died of congestion, at Atlanta, Ga., February 4, 1887. He was the only son of Mr. Charles Kintz, a most worthy and respectable citizen, and his second wife, Clara (*nee* Brady). In his sudden and brief illness he was far away from parents and home, but he was surrounded by the kind friends his many virtues had won during his brief sojourn in this city. Everything was done that affection and medical skill could suggest; but the fiat had gone forth, and after a few hours of suffering he peacefully passed away.

Mr. Kintz was instructed by his parents in the doctrines of the Catholic Church, and he was a devout and consistent member of that religious body during his life. He made such rapid progress in his studies when attending the district schools in his neighborhood, and his character was so admirable, at the age of seventeen he was granted a teacher's certificate, and four years afterwards he was made Superintendent of the Public Schools of Readville, Ohio. While holding this position he became much interested in the study of medicine, and began reading under the instruction of Dr. N T. McTeague. He afterwards read medicine under Dr. Macgruder, at Somerset, and then attended a course of lectures at the Starling Medical College, of Columbus. While there his lungs became feeble, and, thinking a warmer climate might be beneficial, he came to Atlanta last Fall to complete his studies at the Southern Medical College. Had he lived he would have received his diploma from this Institution on the 4th of March last. Mr. Kintz was one of the most exemplary young men we have ever known. Amiable and gentle in disposition, kind and accommodating in manner, he was generally loved and respected by his fellow-students and the Faculty of the College. He was unusually studious and nobly ambitious, and gave promise of reaching high attainments in the loved profession he had chosen. But he is gone from among us in all the prophetic brightness of a future career and, we trust, to that blest land where pain and disappointment are never known. To his parents, brothers and sisters, we tender our heartfelt sympathy, with the hope that into their mourning hearts God will pour the balm of His tender consolation. T. S. P.

